# Selection and dynamics of embryonic stem cell integration into early mouse embryos

**DOI:** 10.1242/dev.124602

**Published:** 2016-01-01

**Authors:** Stoyana Alexandrova, Tuzer Kalkan, Peter Humphreys, Andrew Riddell, Roberta Scognamiglio, Andreas Trumpp, Jennifer Nichols

**Affiliations:** 1 Wellcome Trust-Medical Research Council Cambridge Stem Cell Institute, University of Cambridge, Tennis Court Road, Cambridge CB2 1QR, UK; 2 Department of Physiology, Development and Neuroscience, University of Cambridge, Downing Street, Cambridge CB2 4BG, UK; 3 Division of Stem Cells and Cancer, Deutsches Krebsforschungszentrum (DKFZ), Im Neuenheimer Feld 280, Heidelberg 69120, Germany; 4 Heidelberg Institute for Stem Cell Technology and Experimental Medicine (HI-STEM gGmbH), Im Neuenheimer Feld 280, Heidelberg 69120, Germany

**Keywords:** Pluripotency, Embryonic stem cell, Mouse blastocyst, Chimaera, Live imaging

## Abstract

The process by which pluripotent cells incorporate into host embryos is of interest to investigate cell potency and cell fate decisions. Previous studies suggest that only a minority of the embryonic stem cell (ESC) inoculum contributes to the adult chimaera. How incoming cells are chosen for integration or elimination remains unclear. By comparing a heterogeneous mix of undifferentiated and differentiating ESCs (serum/LIF) with more homogeneous undifferentiated culture (2i/LIF), we examine the role of cellular heterogeneity in this process. Time-lapse *ex vivo* imaging revealed a drastic elimination of serum/LIF ESCs during early development in comparison with 2i/LIF ESCs. Using a fluorescent reporter for naive pluripotency (Rex1-GFP), we established that the acutely eliminated serum/LIF ESCs had started to differentiate. The rejected cells were apparently killed by apoptosis. We conclude that a selection process exists by which unwanted differentiating cells are eliminated from the embryo. However, occasional Rex1^−^ cells were able to integrate. Upregulation of Rex1 occurred in a proportion of these cells, reflecting the potential of the embryonic environment to expedite diversion from differentiation priming to enhance the developing embryonic epiblast.

## Abstract (precis)

**Summary:** Following injection of mouse embryonic stem cells into host embryos, there is preferential and rapid elimination of unwanted differentiating cells by apoptosis.

## INTRODUCTION

Pluripotent stem cells provide a valuable system to explore intrinsic and extrinsic requirements for self-renewal *in vitro*. Murine embryonic stem cells (ESCs) are derived from epiblasts of late blastocysts (Boroviak et al., 2014; Brook and Gardner, 1997; Ying et al., 2008). Their potential to produce all tissues, including gametes, when injected into host embryos defines them as naive pluripotent (Nichols and Smith, 2009). ESCs can be propagated in medium containing foetal calf serum and leukaemia inhibitory factor (LIF) (Smith et al., 1988; Williams et al., 1988). In these conditions, developmentally advanced cells can be distinguished and cultures exhibit heterogeneous expression of markers for naive pluripotency, such as *Nanog*, *Rex1* (*Zfp42*), *Stella* (*Dppa3*), *Pecam1* and *Klf4* (Chambers et al., 2007; Furusawa et al., 2004; Hayashi et al., 2008; Kalmar et al., 2009; Marks et al., 2012; Toyooka et al., 2008). A culture regime was subsequently developed based upon inhibition of the MEK/ERK pathway and GSK3, known as ‘2i’ (Ying et al., 2008). ESCs propagated in 2i exhibit more homogeneous expression of naive pluripotency markers (Nichols and Smith, 2009; Wray et al., 2010). Comparative profiling of ESCs propagated in serum/LIF versus 2i/LIF confirmed these differences (Marks et al., 2012).

Generation of chimaeras from ESCs is used extensively to create transgenic mouse lines (Thomas and Capecchi, 1987) or to test the potency of putative pluripotent stem cells (Bradley et al., 1984). This is generally achieved by providing 8-20 ESCs to a host morula or blastocyst. An inoculum of fewer donor cells tends to produce chimaeras less efficiently (Beddington and Robertson, 1989). A probable explanation of this phenomenon is that only a proportion of the injected cells can integrate into the embryo. In support of this, a maximum of three ESCs per chimaera were observed to produce progeny contributing significantly to the adult animal (Wang and Jaenisch, 2004). Based upon experimental enrichment of ESCs expressing markers of naive pluripotency, it might be assumed that the ESCs permitted to contribute to the embryo are those residing in the naïve state (Furusawa et al., 2004; Toyooka et al., 2008).

The capacity of the morula environment to alter the developmental trajectory of lineage-specified cells isolated from blastocysts was a surprising revelation (Grabarek et al., 2012). Whether the embryonic niche can exercise a similar effect on lineage-priming ESCs is currently unknown. Understanding how the environment can influence exit from pluripotency and its potential reversion is important for the design of *in vitro* differentiation protocols and interpretation of transplantation studies. The recent advances in transgenic reporters and live imaging open the possibility to explore how incoming ESCs incorporate into chimaeras and determine the fate of those that are rejected.

In this study, we exploit two culture regimes: serum/LIF (SL) and 2i/LIF (2iL) to provide ESCs that are more (SL) or less (2iL) heterogeneous for markers of naive pluripotency. ESCs are injected into host embryos at the 8-cell stage. By tracking the process of chimaera formation, spatial and temporal trends for integration or exclusion can be uncovered. We also use a validated destabilised GFP reporter of the zinc finger protein Rex1 (Rex1-GFPd2), which correlates closely with naive pluripotency *in vivo* and *in vitro* (Pelton et al., 2002; Wray et al., 2011). This enables separation of SL-cultured ESCs into naive pluripotent (Rex1^+^) and developmentally advanced (Rex1^−^) populations prior to injection. In addition, GFP fluorescence enables assessment of the pluripotency status of integrating or excluded cells during chimaera formation.

Our results uncover some interesting phenomena. Firstly, a large proportion of SL-cultured ESCs is dramatically eliminated by apoptosis within the first few hours after injection. Coincidentally, surviving ESCs appear to undergo compensatory proliferation. Secondly, 2iL-cultured ESCs continue to proliferate throughout the experiment, but undergo increased apoptosis during the second day of culture, in concert with the second lineage segregation event of the host embryo. Finally, although the majority of eliminated cells appear to have begun exit from pluripotency, Rex1^−^ cells can occasionally upregulate GFP expression during development, but this is not a conditional prerequisite for integration into the epiblast.

## RESULTS

### ESCs cultured in 2iL out-perform those from SL conditions during chimaera formation

**Fig. 1.**
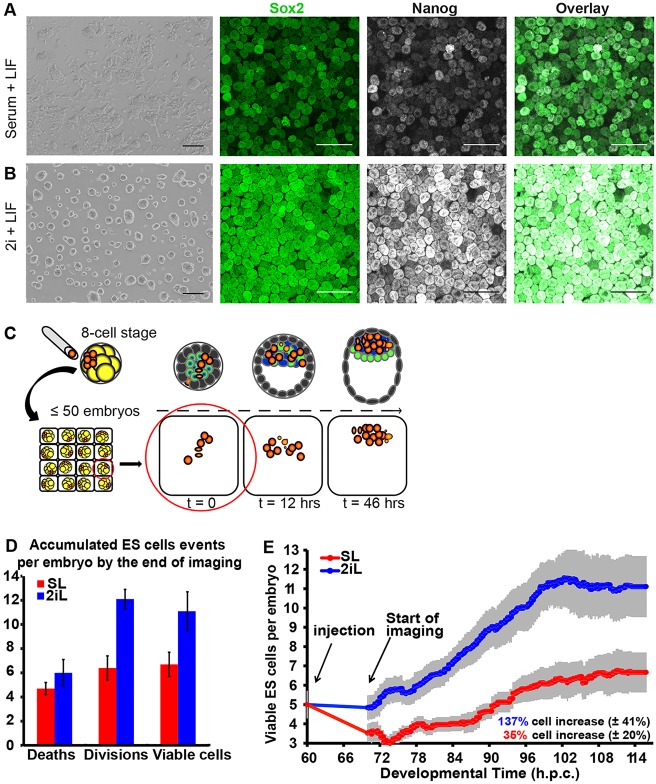
**Comparison of ESCs cultured in conventional versus ground-state conditions.** Morphology and immunohistochemistry of ESCs cultured for 2 days in (A) conventional, serum/LIF (SL) or (B) ground-state, 2i/LIF (2iL) conditions. Left panels: bright field; second and third panels: immunoreactivity to Sox2 (green) and Nanog (white), respectively; right panels: overlay of Sox2 and Nanog. (C) Scheme for the experimental strategy: 8-cell embryos were injected with fluorescently labelled ESCs and chimaeras transferred to an immobilising grid for live imaging for 2 days. (D) Bar plot of the average numbers of ESC deaths, divisions and resulting viable ESCs by the end of captured development. (E) Plot of the average numbers of viable ESCs per embryo over time (hpc). Grey bars reflect s.e.m. between the curves of the five embryo groups (profiles per embryo injected with 3-7 ESCs). See Table S1 for full data. Scale bars: 100 µm in A,B.

To test the hypothesis that ESCs in the state of naive pluripotency preferentially integrate into chimaeras, we used two alternative culture conditions. ESCs propagated in SL for at least four passages exhibited a substantial level of heterogeneity, both morphologically and by immunohistochemistry (Fig. 1A). Those expanded using 2iL formed more compact, rounded colonies and a higher proportion expressed pluripotency markers Sox2 and Nanog (Fig. 1B).

ESCs labelled with ubiquitous tdTomato-H2B (Morgani et al., 2013) were used to facilitate tracking in chimaeras. This reporter localises to chromatin and therefore serves as an ideal identifier of nuclear fragmentation associated with cell death and separation of chromosomes during mitosis (Fig. S1A,B). Each pre-compacted 8-cell embryo was injected with 3-7 ESCs (Table S1); pooled data from two experiments were separated into five groups based on the number of injected ESCs per embryo (3, 4, 5, 6 or 7 cells). Embryos isolated at mid-day on the third day after mating were assumed to approximate 60 h post coitum (hpc). They were tracked by live imaging from about 10 h post-flushing until the late blastocyst stage, 36-38 h later (Fig. 1C). The emergence of morphology consistent with cell death or division was recorded every 20 to 30 min. The first 10 h after flushing was not recorded to minimise fluorescence exposure and promote healthy development. However, analysis of movies revealed condensed and fragmented ESC nuclei assumed to have undergone apoptosis at the start of imaging (Fig. S1C), indicating that cell death occurred soon after injection. Apoptotic cell counts during 60-70 hpc were extrapolated by adding ESC deaths detected before imaging. To score the incidence of death, division and location of injected ESCs, chimaeras were filmed in 4D (three physical dimensions and time). Using Fiji (ImageJ) TrackMate manual tracking, a total of 46 embryos across two experiments were analysed: 18 SL-injected embryos (producing 16 chimaeras) and 28 2iL-injected embryos (28 chimaeras).

Analysis of time-lapse movies produced a dataset containing all ESC deaths and divisions scored temporally for each embryo. The numbers of viable ESCs were determined per embryo (Fig. 1D and Table S1). Injected ESCs and their progeny are referred to as ‘ESCs’ hereafter, for simplification. Comparison of the percentage increase of viable 2iL and SL ESCs across embryos at the end of culture revealed a statistically significant difference (Table S1; *P*=0.0265). Embryos injected with 2iL ESCs incorporated a higher number of viable ESCs (137.4±41.3%; mean±s.d.) compared with those injected with SL ESCs (34.9±20.2%; Fig. 1E). The survival rate of 2iL ESCs within the embryo remained significantly higher compared with SL ESCs for the duration of recorded development (Fig. 1E; *P*<0.0001).

### SL ESCs exhibit substantial cell death within hours of injection

**Fig. 2.**
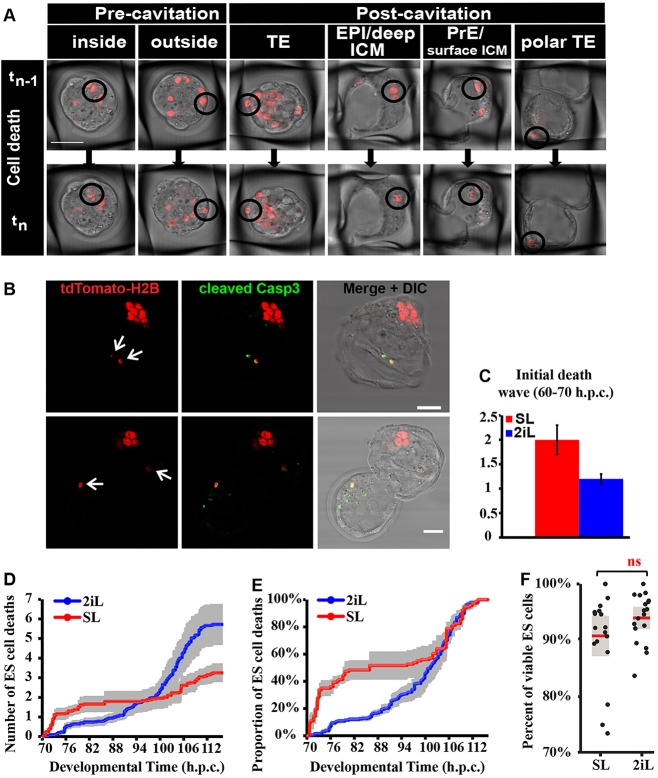
**Differential ESC death dynamics during embryonic development.** (A) Selected progressive snap shots of ESC death events, visualised by disintegration of nuclei during live imaging. (B) ESC nuclear fragments (red) colocalise with cleaved caspase 3 (Casp3, green) in two representative embryos injected with SL ESCs. (C) Bar plot of average numbers of ESC deaths accumulated in the first ∼10 h of development. See Table S2 for full data. (D) Accumulation in developmental time of average number of ESC deaths per embryo. (E) Cumulative distribution plot (CDF) of the ESC deaths per embryo, showing temporal distribution of accumulated ESC deaths from total ESC deaths per embryo. Grey bars reflect s.e.m. between the five curves, where each curve is the temporal profile per embryo injected with 3-7 ESCs. (F) Results of the *in vitro* control experiment for cell death: 300,000 ESCs plated per well in SL or 2iL (18 wells per condition) and counted 24 h later. Each black dot displays percentage viable ESCs of total cells counted per well. Each box plot is overlaid with the raw data, distributed along *x*-axis for clarity. Red line represents average value and grey box 1 s.d. *P*=0.1372 [non-significant (ns)]. Scale bars: 50 µm in A; 30 µm in B.

The differential survival of SL versus 2iL ESCs must arise from quantitative differences in cell death, proliferation, or both. To ascertain the underlying cause, the dynamics and distribution of ESC death was investigated further. Incidence of ESC death and division differed between the five embryonic groups injected with 3, 4, 5, 6 or 7 ESCs; however, no consistent correlation between the number of starting ESCs and their subsequent rate of proliferation or elimination could be assigned (Figs S2-S4). Therefore, for the remaining analysis the behaviour of ESCs across the five individual embryo groups was averaged. The morphological manifestation of nuclear fragmentation or condensation resulting in eventual loss of the cell (Fig. 2A) is characteristic of apoptosis (Fig. S1A). Immunohistochemistry of ESC nuclear fragments from apoptotic events revealed reactivity for cleaved caspase 3 (Fig. 2B). SL ESCs displayed an acute ‘death wave’ within 10 h of injection (Fig. 2C); 27.6% of the injected SL ESCs died within this time window, compared with only 4.3% of 2iL ESCs (Table S2). The transcriptional profile of ESCs in 2iL closely resembled that of the preimplantation epiblast, whereas SL ESCs are generally more divergent (Boroviak et al., 2014). By the end of culture, the death rate for 2iL ESCs was higher than for SL ESCs (Fig. 1D), but this was a consequence of increased survival during the first 10 h. ESCs from 2iL did, however, exhibit a peak of elimination from around 100-110 hpc (Fig. 2D). Thus, the incidence of ESC death accumulation is dynamically different between the two groups (*P*<0.0001), reflecting divergent properties of donor cells imposed by their culture history.

To evaluate the incidence of ESC death, we examined the cumulative distribution function (CDF). This represents the temporal distribution of the accumulated ESC deaths per embryo. This analysis showed that SL ESC deaths accumulated at a strikingly higher rate early in chimaera formation, whereas such behaviour was not apparent in 2iL ESCs (Fig. 2E). Approximately half (48%) of the deaths for SL ESCs occurred by 82 hpc compared with only 12% for 2iL ESCs. After the initial death wave, SL ESCs exhibited lower levels of death (Fig. 2D,E). Interestingly, at the blastocyst stage (100-110 hpc) an increase in death was observed for both conditions (Fig. 2D,E). This second death wave coincided with the apoptosis previously reported in ICM cells during sorting of the host epiblast and primitive endoderm (PrE) lineages, whereby ‘mis-positioned’ cells might be eliminated (Morris et al., 2010; Plusa et al., 2008). Asserting the importance of the embryonic environment in the choreography of cell mortality, no significant difference in cell death frequency was observed between SL and 2iL ESCs after 24 h of culture *in vitro* (Fig. 2F). We propose that the embryo may actively eradicate ‘unsuitable’ ESCs based on their differentiation status.

### SL ESCs undergo compensatory proliferation during the early phase of cell death

**Fig. 3.**
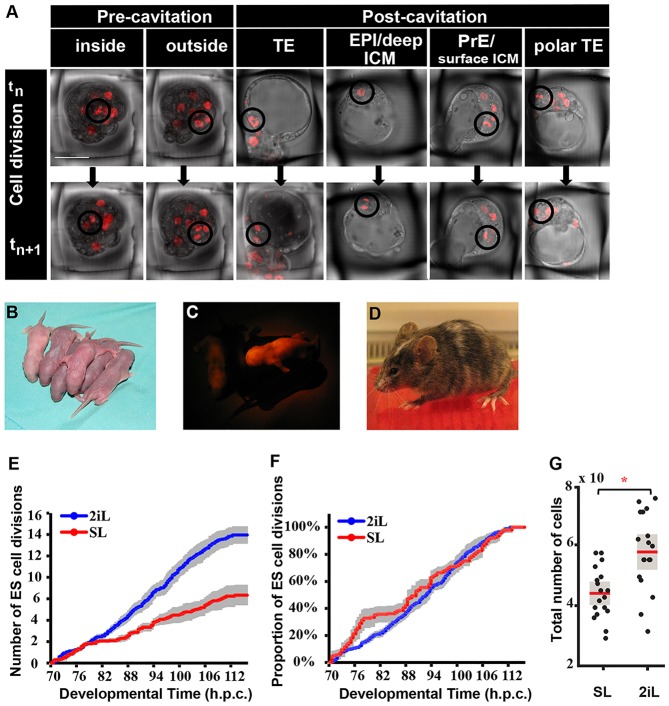
**Differential ESC division dynamics during embryonic development.** (A) Selected progressive images of ESC division events, visualised by condensation of fluorescently tagged chromatin and appearance of two smaller cells at the next time point during live imaging. (B) Pups born from injected embryos transferred to recipients after imaging for 40 h. (C) Red fluorescent image of chimaeric pups. (D) Adult chimaera from pup shown in C. (E) Accumulation in developmental time of average ESC divisions per embryo. (F) Cumulative distribution plot (CDF) of ESC divisions per embryo, exhibiting temporal distribution of accumulated ESC divisions from total per embryo. Grey bars reflect s.e.m. between curves, where each curve is the temporal profile per embryo injected with 3-7 ESCs. (G) Result of *in vitro* control experiment (shown in Fig. 2F) with respect to cell division; each black dot displays total number of ESCs in a single well in SL or 2iL 24 h after plating. Total cell number includes viable ESCs plus non-viable (apoptotic) cells. The total number of ESCs is significantly different in SL versus 2iL groups, *P<0.0001. Scale bar: 50 µm in A.

ESC mitoses within chimaeras were scored (Fig. 3A, Fig. S1B). That development of host embryos was not irreparably damaged by the imaging procedure was confirmed by birth of live animals following transfer of filmed embryos to recipient mice (Fig. 3B-D). From 17 embryos injected with SL-cultured cells and imaged for 39.5 h, six mice were born, two of which exhibited red fluorescence. This was lower than expected, based on the number of chimaeras at the blastocyst stage (16/18). Therefore, we cannot eliminate the possibility that repeated exposure to fluorescent excitation during the culture period adversely affected the injected ESCs in postimplantation stages.

The fraction of newly generated cells from the total injected was calculated. The number of cell divisions per embryo was higher for 2iL than SL ESCs (*P*=0.0056) (Fig. 3E, Table S3). Normalisation of the mitotic accumulation by the total number of ESC division events in each group (CDF) revealed fluctuations of the proliferation rate for SL ESCs (Fig. 3F). A 24 h *in vitro* chase experiment indicated that 13.4% more ESCs had divided in 2iL compared with in SL (Fig. 3G; *P*=0.00055). Hence, the comparative reduction in SL ESC divisions throughout chimaera formation is likely to be caused by their overall reduced proliferation rate, in combination with the persistence of fewer cells after the initial wave of elimination (Fig. 2C, Table S2).

SL ESCs displayed a transient increase in division rate during the early phase of integration, peaking between 70 and 82 hpc (Fig. 3F), coinciding with the first ESC elimination phase (Fig. 2E). This suggests that two population states emerge from the pool of injected SL ESCs: one directed to undergo apoptosis and another that is permitted to propagate. This behaviour is characteristic of the ‘compensatory proliferation’ observed in competition assays, where ‘more fit’ cells undergo a surge in division, which is interpreted as a means to compensate for elevated cell death of ‘less-fit’ cells (Sancho et al., 2013). By contrast, 2iL ESC divisions occurred uniformly throughout recorded development (Fig. 3F).

### Spatial distribution of cell death and proliferation during chimaera integration

**Fig. 4.**
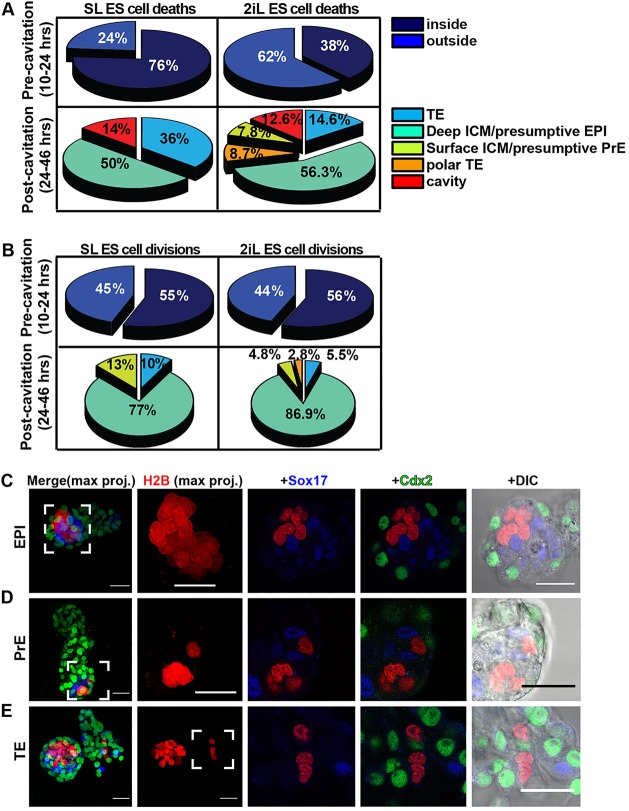
**Localisation of** **ESCs during chimaera formation.** (A) Pie charts displaying percentage of SL or 2iL ESC deaths recorded at different locations in embryos. Top panels depict ESC localisation during day 1 of development prior to cavitation; bottom panels display day 2. Percentages are exclusive of deaths or divisions not clearly assigned to an embryonic location because of restricted visibility (20-30% of events). (B) Pie charts displaying percentages of SL or 2iL ESC divisions recorded at different locations in embryos, as for cell death events in A. (C-E) Representative images of ESCs localising to epiblast (C, 30/30), primitive endoderm (D, 2/30) or trophectoderm (E, 3/30) in late blastocyst chimaeras. Two left panels are maximum projections; remaining images are single planes. tdTomato-H2B, red; Sox17, blue; Cdx2, green. Scale bars: 30 µm in C-E.

Each embryo was analysed at every time point for the location of ESC death and division events (Fig. 2A and Fig. 3A). Analysis of embryos injected with either SL or 2iL ESCs revealed that donor cell death occurred in both the inner and outer area of the embryo during the first day of imaging, with the majority localising to the inside for SL and vice versa for 2iL (Fig. 4A). During subsequent development, it was restricted predominantly to the epiblast and TE lineages for SL ESCs, but was observed in all lineages for 2iL ESCs, with the majority occurring in the epiblast (Fig. 4A).

The distribution of divisions for both SL and 2iL ESCs was similar during the first day (Fig. 4B). No division was observed for any cell that did not integrate. After cavitation, the majority of divisions tended to occur predominantly in the epiblast for both 2iL and SL ESCs (Fig. 4B).

### Positional fate of integrated ESCs

Following filming, chimaeras were processed for immunohistochemistry. The majority contained progeny of injected ESCs solely in the epiblast lineage (Fig. 4C). Although 3/30 (two SL and one 2iL) contained a total of 7 ESCs located in the PrE domain (Fig. 4D), none of them expressed the early PrE marker Sox17. Similarly, 7 ESCs were found in the TE region of 2/30 chimaeric embryos (2iL), but no Cdx2 expression was apparent (Fig. 4E). These results suggest that viable progeny of ESCs do not acquire extraembryonic lineage identity in response to environmental stimuli during development of preimplantation chimaeras, at least within the context of this study.

### Eradication of injected ESCs coincides with progression towards differentiation

Markers of naive pluripotency characteristic of the E4.5 epiblast are more specifically enriched in 2iL-cultured ESCs, compared with SL ESCs, which tend to cluster towards the postimplantation epiblast (Boroviak et al., 2014). We hypothesise that the SL cells succumbing to elimination from chimaeras are developmentally more advanced. To test this, we imaged embryos injected with purified ESCs expressing high or low levels of the pluripotency marker Rex1. Previous work demonstrated that in SL, ESCs with a GFP knock-in at the *Rex1* locus constitute a mixture of Rex1-GFP^high^ and Rex1-GFP^low^ populations that can be isolated by flow cytometry (Marks et al., 2012). The Rex1^−^/Oct4^+^ population, comprising up to 50% cultured ESCs, was suggested to resemble early postimplantation epiblast (Marks et al., 2012; Toyooka et al., 2008).

**Fig. 5.**
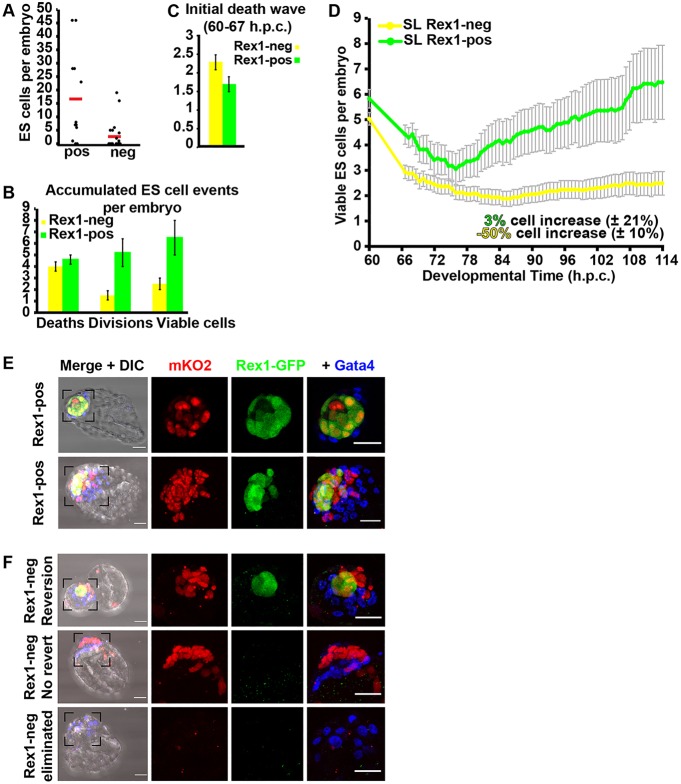
**ESCs commencing differentiation are preferentially eliminated from host embryos.** (A) Number of ESCs, sorted from SL cultures for presence (pos) or absence (neg) of Rex1-GFP, incorporated into injected embryos at the blastocyst stage (113 hpc). 8/22 Rex1^−^ chimaeras and 10/12 Rex1^+^ chimaeras are displayed as black dots above baseline. A second experiment, conducted with mKO ESCs, is presented in Fig. S5. Each boxplot is overlaid with raw data, where each black dot represents data from a single embryo; red line shows mean value. (B) Bar plot for average numbers of ESC deaths, divisions and resulting viable ESCs by the end of culture (see Table S1). (C) Bar plot of average numbers of ESC deaths accumulated in first 7 h of development; for full details, see Table S2. (D) Plot of average numbers of viable ESCs per embryo; grey bars reflect s.e.m. between embryos. (E) Immunohistochemistry of embryos injected with mKO ESCs sorted for high (Rex1^+^) GFP expression after 2 days of culture (see Fig. S6 for details of sorting). 16/17 chimaeras exhibited a mixture of positive and negative Rex1-GFP cells, ranging from a single Rex1^−^ cell in the epiblast (top embryo, single planes) to a significant proportion of the epiblast (bottom embryo, max projection). (F) Immunohistochemistry images (max projections) of three representative embryos for the three outcomes generated from Rex1^−^ ESC injections. Top panels show Rex1^+^ epiblasts in embryos injected with Rex1^−^ ESCs (4/19). Middle panels illustrate chimaeras from Rex1^−^ ESCs not expressing GFP (7/19). Bottom panels show embryos that lost Rex1^−^ ESCs during culture (8/19). Scale bars: 30 µm in E,F.

Rex1-GFP^low^ (Rex1^−^) and Rex1-GFP^high^ (Rex1^+^) ESCs were separated by fluorescent cell sorting (top and bottom 5%, respectively) before injection into embryos (3-8 cells per embryo), which were either incubated or imaged for 2 days. Subsequent analysis revealed that embryos injected with Rex1^−^ ESCs form chimaeras with significantly fewer ESC progeny compared with those generated from Rex1^+^ ESCs (Fig. 5A, Table S4). In each of two separate experiments, a proportion of embryos injected with Rex1^−^ ESCs was found to be non-chimaeric (14/22 and 8/19; Fig. 5A, Fig. S5), whereas Rex1^+^ ESCs contributed robustly (10/12 and 17/17; Fig. 5A, Fig. S5).

Fig. 5B and Table S1 show total numbers of ESC deaths, divisions and viable donor cells per injected embryo. Rapid elimination of around half the donor cells was observed within the first 7 h of culture in Rex1^−^ ESC-injected embryos (Fig. 5C, Table S2), whereas only 14% of Rex1^+^ ESCs were lost during this period. By the late blastocyst stage, Rex1^+^-injected embryos contained significantly more viable ESCs than the Rex1^−^ group (*P*=0.0021; Fig. 5B,D), because Rex1^−^ ESCs were more extensively eliminated and divided less frequently throughout the experiment (Table S3; Fig. 5D). This corroborates the hypothesis that developmentally advanced (Rex1^−^) ESCs are selectively eliminated during chimaera formation.

### Rex1^−^ ESCs can upregulate Rex1 *in vivo*

Inspection of chimaeras from Rex1^+^ ESC injections revealed that 16/17 epiblasts contain a mixture of Rex1-GFP^−^ and Rex1-GFP^+^ ESC progeny (Fig. 5E). The number of Rex1-GFP^−^ cells ranged from a single cell (Fig. 5E, top panel) to a significant proportion of the ESC-derived epiblast (Fig. 5E, bottom panel). This is consistent with downregulation of Rex1 at the onset of implantation and exit from naive pluripotency. Chimaeras generated from Rex1^−^ ESC injections occasionally (4/19 cases) exhibited Rex1-GFP fluorescence at the blastocyst stage (Fig. 5F, top panel). In the remaining Rex1^−^ chimaeras (7/19), donor cell progeny not expressing Rex1 persisted in the epiblast (Fig. 5F, middle panel). These cells may represent ‘epiblast’ that has advanced beyond the Rex1^+^ stage. In 8/19 Rex1^−^ ESC-injected embryos, no surviving ESC progeny were detected (Fig. 5F, bottom panel).

The 4 Rex1-GFP^+^ chimaeras from 19 Rex1^−^ ESC-injected embryos are unlikely to be solely attributable to contamination of the fluorescent cell sorting prior to injection, because only 0.64% total contaminants were detected in simultaneous purity checks of 10,000 cells from the injected population (Fig. S6). To investigate potential embryo-induced Rex1 upregulation further, we injected bi-allelic Rex1^−^ ESCs into 8-cell-stage embryos (*n*=19) and imaged for 2 days (Fig. S7A-D). Live chimaera imaging showed that Rex1^−^ to Rex1^+^ conversion occurred at low rates (2/19, Fig. S7A,B, Table S5). As one control, 5 ESCs were explanted per well of a 96-well plate from each sorted population and cultured for 12 days in SL (Fig. S7E,F). Although the results indicated that 6/60 Rex1^−^ ESCs formed small colonies, GFP was not observed, in contrast to the majority (21/24) originating from Rex1^+^ ESCs (Fig. S7E). Consistent with this, it was reported that Rex1^−^ ESCs cannot produce undifferentiated colonies in SL culture, even when plated in high density from less-stringent Rex1-GFP^−^ cell sorting; however, a few undifferentiated colonies emerged in 2i (Marks et al., 2012). We therefore plated Rex1^−^ sorted cells into 2iL at high and low density (Fig. S7F,G). To increase the potential for reversion, they were also plated on mitotically inactivated murine embryonic fibroblasts (Hayashi et al., 2008). Rex1^−^ ESCs could form Rex1^+^ colonies within 7 days of culture in 2iL at low frequencies (0.1-1.4%; Fig. S7F,G). Comparing this rate with chimaera experiments (Fig. S7F, Fig. 5A and Table S5), we observed more Rex1 reversion within the embryo (3.3%), although the difference was not statistically significant.

### Elimination of ESCs is independent of differential c-Myc levels in preimplantation chimaeras

**Fig. 6.**
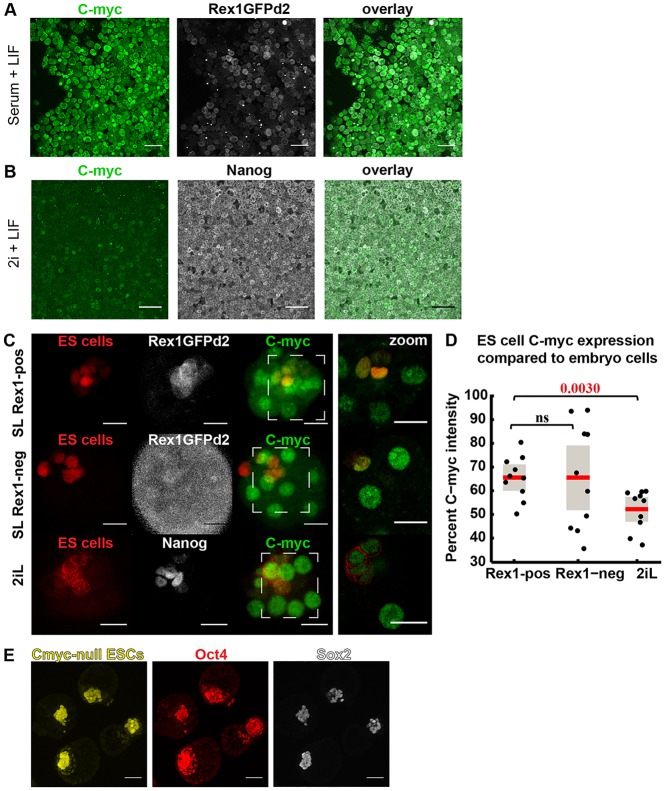
**Expression of** **c-Myc in ESCs and chimaeras.** (A) Immunohistochemistry of SL ESCs and (B) 2iL ESCs for c-Myc (green), Rex1-GFPd2 (white) or Nanog (white). SL ESCs are mKO2 Rex1-GFP, whereas 2iL ESCs are membrane-bound CFP Confetti. (C) Representative chimaeras from SL Rex1^+^ (top panels, *n*=10), SL Rex1^−^ (middle panels, *n*=10) and 2iL (bottom panels, *n*=10) ESC injections. Left-hand images show maximum projection of whole embryos; right-most images show higher magnification of single planes. (D) Ratio of c-Myc expression in ESCs relative to host cells in chimaeras. Each black dot represents relative expressions levels in a single chimaera. Each box plot is overlaid with raw data distributed along *x*-axis for clarity; red lines indicate average values; grey box is s.e. Intensity of fluorescence for all ESCs and host cells were measured manually on ImageJ (see the Materials and Methods). (E) Representative chimaeras whose ES-derived epiblasts express naive pluripotency markers from 13/14 embryos injected with EYFP c-*Myc* null ESCs. Scale bars: 50 µm in A,B,E; 20 µm in C.

Previous studies demonstrate that postimplantation epiblast cells with low c-Myc expression preferentially undergo apoptosis (Clavería et al., 2013; Sancho et al., 2013). Cell selection is apparently triggered by heterogeneity of c-Myc expression between neighbouring cells in the postimplantation epiblast and in ESC cultures following several days in conditions driving exit from naive pluripotency and assumption of primed pluripotency. To determine whether differential c-Myc expression is responsible for elimination of differentiating ESCs during chimaera formation, embryos were injected, cultured for 7-10 h (during the wave of ESC elimination) and inspected for expression of c-Myc and pluripotency markers. Intensity of marker expression was quantified using ImageJ and Volocity. c-Myc protein was observed in both 2iL and SL ESCs (Fig. 6A,B), but at levels consistently below those in host embryos (Fig. 6C). Furthermore, c-Myc expression was lower for 2iL than SL ESCs *in vitro* and in chimaeras (Fig. 6A-D). We saw no consistent correlation between c-Myc and Rex1 or Nanog levels in ESCs (Fig. 6C), or any obvious difference between chimaeras of sorted Rex1^−^ or Rex1^+^ SL ESCs (Fig. 6D). To determine whether Nanog correlates with c-Myc expression in 2iL chimaeras, the intensity of Nanog and c-Myc of each ESC was measured as a percentage of the brightest ESC (Fig. S8). Only a weak correlation could be detected (Pearson correlation *r*=0.481, *P*=0.001). Further evidence that differential c-Myc expression between donor ESCs and the embryo is unlikely to play a role in ESC selection in this context was provided by generation of chimaeric blastocysts from embryos injected with c-*Myc* null ESCs (Fig. 6E).

### Downregulation of pluripotency factors may mark cells for elimination

**Fig. 7.**
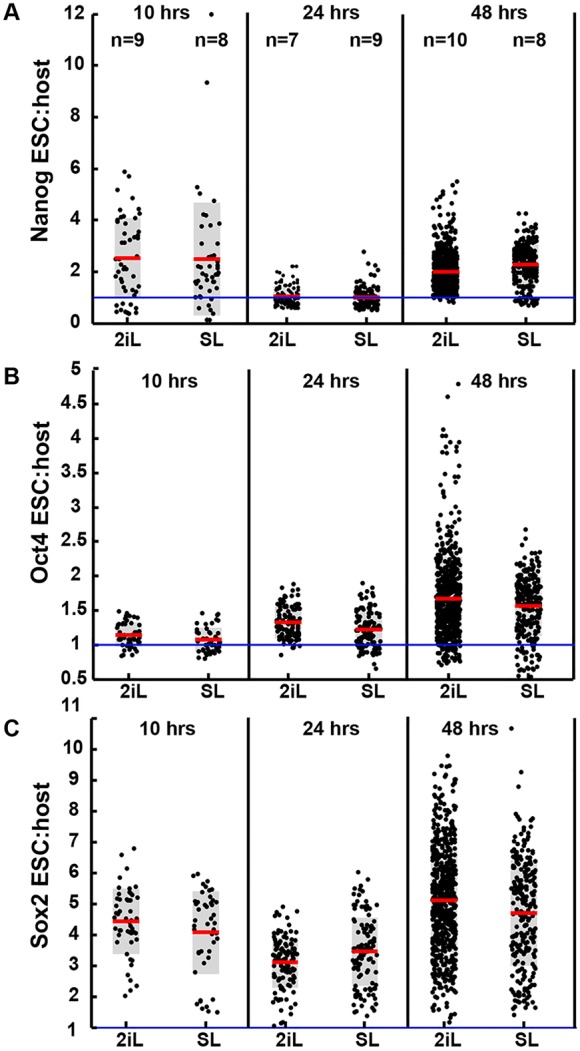
**Ratio of marker expression in donor ESCs relative to embryonic cells.** Intensity levels for expression of (A) Nanog, (B) Oct4 and (C) Sox2 in ESCs relative to average expression level for host embryo cells. Values at the top (A) indicate number of chimaeras inspected at different time points. Each black dot represents expressions levels for a single ESC relative to the host. Each box plot is overlaid with data distributed along *x*-axis for clarity; red lines indicate mean values, grey box is s.e. Intensity of fluorescence was detected using Volocity (see the Materials and Methods).

To challenge further the hypothesis that differentiating cells are selected for elimination by the host embryo, we questioned whether reduced expression of pluripotency markers could presage elimination of donor ESCs. We quantified expression of Nanog, Oct4 and Sox2 at 10 h, 24 h and 48 h post-injection of ESCs (Fig. 7). Expression appeared higher in most ESCs compared with host cells, at all time points. However, we noted a comparative decrease in average expression of the naive pluripotency markers, Nanog and Sox2 at 24 h, which coincides with the onset of segregation of epiblast from PrE in the host embryo and the second wave of cell death of injected ESCs.

## DISCUSSION

Challenging the regulative capacity of the developing mouse embryo by provision of supernumerary cells affords a means to explore the mechanisms by which cells are incorporated or rejected. That a selection procedure operates during this process has been retrospectively inferred from previous studies by inspecting fixed chimaeric embryos and adult tissues (Saburi et al., 1997; Wang and Jaenisch, 2004). To attempt to uncover a mechanism for this phenomenon, we used confocal live imaging to compare the behaviour of populations of largely undifferentiated (2iL) and mixtures of undifferentiated and developmentally advanced (SL) ESCs during integration. Strikingly, we recorded a dramatic and reproducible wave of cell death within the first few hours following injection of SL-cultured ESCs, which was not seen in 2iL chimaeras. Subsequently, more 2iL donor cells persisted (Fig. 1D,E), which is consistent with the idea that the developmentally advanced cells from SL cultures are preferentially eliminated. However, cell death during the second day of culture, particularly in 2iL chimaeras, increased dramatically, coincident with the onset of epiblast and PrE sorting in the host embryo (Fig. 2D). We speculate that this may result from overcrowding caused by a combination of reduced cell death during day 1 and increased division throughout the experiment in the 2iL inoculum (Fig. 2D and Fig. 3E,G). Furthermore, as development progresses in the absence of inhibitors, some 2iL ESCs may be exiting naive pluripotency, leading to higher elimination during day 2. This phenomenon is reflected in the relative downregulation of the naive pluripotency markers Nanog and Sox2, compared with the core pluripotency marker Oct4, that is observed in some cells following 24 h of culture (Fig. 7), which coincides with the onset of the second death wave (Fig. 2D,E).

**Fig. 8.**
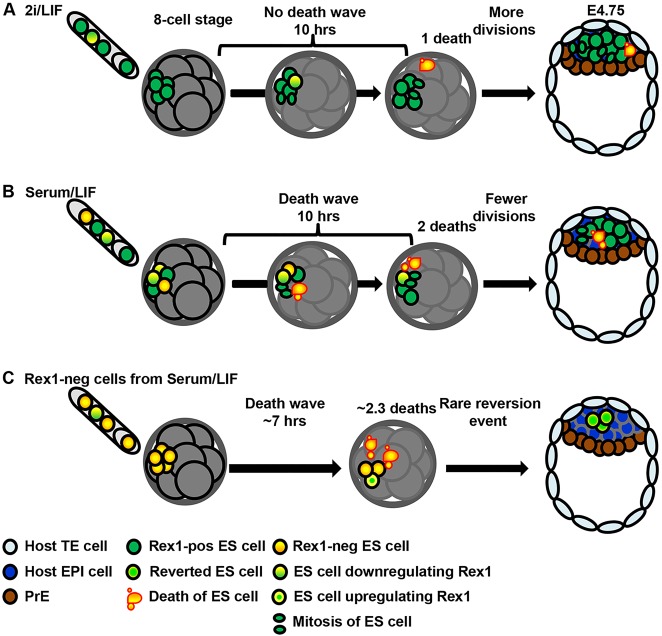
**Model for incorporation of ESCs into early mammalian embryos.** (A) ESCs cultured in stringent ground state conditions (2i/LIF) represent a nearly homogeneous culture of undifferentiated cells. Most injected cells incorporate and contribute to the epiblast. (B) ESCs cultured in conventional conditions (serum/LIF) represent a heterogeneous culture of undifferentiated and more developmentally advanced cells. In the embryonic environment, a wave of elimination is observed within a few hours. (C) Rex1^−^ ESCs are acutely eliminated when placed into the embryonic environment. The outcome by E4.75 is either complete loss of ESC progeny or incorporation of Rex1^−^ ESCs into the blastocyst (at variable frequencies), or in rare cases (depicted here), upregulation of Rex1 in one or more cells during the late blastocyst stage.

A system of selective cell elimination in the early post-implantation epiblast is described to depend upon differential c-Myc expression (Clavería et al., 2013; Sancho et al., 2013). However, the existence of such a process has not been shown in the preimplantation embryo. Our results suggest that a selection mechanism exists in preimplantation development to eliminate preferentially more advanced cells by pro-apoptotic instruction (Fig. 8). However, this could not be attributed to differential c-Myc expression between host embryo and donor ESCs. Our results are consistent with previous work in which cell competition was not observed between naive pluripotent ESCs, but realised only once they had transited to the primed state (Sancho et al., 2013). The exact mechanism for donor ESC elimination by the host embryo remains to be elucidated.

We further tested our hypothesis that acutely eliminated cells in the SL inoculum are those in the process of exiting naive pluripotency by using ESCs expressing destabilised GFP under the control of Rex1. Although most incorporating ESCs were Rex1^+^, a proportion of Rex1^−^ ESCs was also detected in chimaeras generated from sorted Rex1^−^ ESCs (7/19 embryos; Fig. 5F, middle panel; 8/22, Fig. 5A and 11/20, Fig. S5). These cells were also Nanog^−^ (not shown), so may be representative of peri-implantation epiblast, destined to survive and differentiate along with the host epiblast. The apparently healthy nuclear morphology and substantial number of donor cells present after the culture period is consistent with successful integration into the host epiblast. Alternatively, these cells may have been fated for elimination or colonisation of extra-embryonic lineages, if development had been prolonged. GFP was detected in 16% of cultured Rex1^−^ ESC-injected embryos (Fig. 5; Fig. S7). This represents at least 3.3% of the total number of donor Rex1^−^ ESCs. Since the maximum yield of GFP^+^ cells from plating Rex1^−^ cells *in vitro* was only 1.4% (Fig. S7F), we infer that upregulation of GFP within chimaeras may occur by virtue of the permissive capacity of the embryonic environment to enhance the developmental potential of donor cells, as previously shown (Grabarek et al., 2012).

In conclusion, our novel live imaging approach to study chimaera formation reveals that the culture history experienced by ESCs before transfer into the embryonic environment strongly influences their subsequent fate. ESCs previously grown in 2iL, reported to be the closest *in vitro* relative to the preimplantation epiblast (Boroviak et al., 2014), integrate efficiently into host embryos, whereas the embryo rapidly eliminates most unwanted, developmentally more advanced cells. Interestingly, a minority of Rex1^−^ cells can become incorporated into host embryos, with or without concomitant upregulation of the GFP reporter. This revelation emphasises the importance of rigorously determining the potential of living tissue to regulate the behaviour of transplanted cells.

## MATERIALS AND METHODS

Experiments were performed in accordance with EU guidelines for the care and use of laboratory animals, and under the authority of appropriate UK governmental legislation. Use of animals in this project was approved by the Animal Welfare and Ethical Review Body for the University of Cambridge. Relevant Home Office licences are in place. All mice were maintained under a 14 h light:10 h dark cycle with food and water supplied *ad libitum*.

### ESC lines

ESCs expressing tdTomato-H2B (Morgani et al., 2013) were used for most experiments. For visualisation of naive pluripotency, these were electroporated with *Rex1*-*Gfpd2* construct (Marks et al., 2012; Wray et al., 2011). An alternative Rex1 reporter line (mKO2 Rex1-GFP) derived from *Rex1-Gfpd2* homozygous embryos transfected with Kusabira Orange was kindly provided by Carla Mulas (Wellcome Trust-Medical Research Council Cambridge Stem Cell Institute). c-*Myc* null ESCs (clone 11: *c-myc^del/del^; N-myc^flox/flox^*) were derived from ES-D3 cells (R.S., unpublished results). Null cells were generated by transient transfection with Cre recombinase and single clone selection; cells constitutively express EYFP under the *Rosa26* promoter. Confetti ESCs were derived from R26R-Confetti embryos (Snippert et al., 2010), activated by Tat-Cre recombinase treatment and single clones selected.

### ESC culture

ESCs were routinely maintained on 0.1% gelatin-coated plates. Passaging by trypsinisation of ESCs grown in SL or 2iL was synchronised. Generally, cells were plated 300,000 per well in 6-well plates (Corning Life Sciences) with medium changes every 2 days. Culture medium for 2iL comprised N2B27 (Stem Cells NDif N2B27) supplemented with MEK inhibitor PD0325901 (1 μM, Stemgent), GSK3 inhibitor CH99021 (3 μM, Stemgent) and mouse LIF (25 ng/ml, produced in-house). SL ESCs were cultured in Glasgow minimal essential medium (GMEM, Sigma) supplemented with 10% foetal calf serum, 1 mM sodium pyruvate, 100 μM 2-mercaptoethanol, 1× nonessential amino acids and mouse LIF (10 ng/ml). Chromosome counts were performed as described previously (Kawaguchi et al., 2010).

### Generation of chimaeras

Embryos were harvested from F1 (C57BL/6×CBA) or C57BL/6×F1 crosses. Females were selected by morphological identification of oestrus (Champlin et al., 1973). Detection of a copulation plug on the following day confirmed mating. Embryo staging was based on the assumption that mating occurred at midnight, so that at 12 noon the next day embryos are assigned E (embryonic day) 0.5 or 12 hpc. E2.5 (60 hpc) embryos were flushed from oviducts in M2 (Sigma) and cultured in BlastAssist (Origio) under embryo-tested mineral oil (Sigma) at 37°C and 7% CO_2_ in air. ESCs (3-8) were injected via a laser-generated perforation in the zona pellucida using XYClone (Hamilton Thorne Biosciences). For experiments comparing naive pluripotent versus differentiating donor cells, ESCs were sorted for Rex1-GFPd2^high^ (top 5% of population) or Rex1-GFPd2^low^ (bottom 5% of population) expression using a Beckman Coulter MoFlo high-speed sorter immediately before injection.

### Time-lapse image acquisition of chimaera development

Injected embryos were transferred to the environmental chamber of the spinning disk microscope (Andor Revolution XD System with a Nikon Eclipse Ti Spinning Disk) and imaged for 2 days. Twenty-one *z*-stacks per time step (20 or 30 min) were taken, with two channels (567 nm excitation for ESC visualisation, and bright field). Temperature (37°C), CO_2_ concentration (7%) and fluorescence exposure (148 ms of 567, 300 ms bright field) were standardised. Prior to each imaging experiment, the incubation chamber (Oko Lab) was allowed to stabilise to 37°C. The CO_2_ concentration was generated by an active mixer (Life Imaging Sciences) and humidified before supply to the sample. Embryos were immobilised using a 118×118 µm polyester mesh (Plastok Group) in a glass-bottomed dish (MatTek Corporation). An Andor 85 camera recorded images with magnification through a Plan Fluor 40×/1.3 NA oil lens. Each experiment was set up using Andor IQ Software. A multi-position map was created: every embryo was manually assigned an *x-y-z* location at its centre and visited (starting from the upper-most plane) by the 40× lens at each time point of data acquisition. Channels were sequentially acquired per *z*-section. Each image collected data in 502×501 (width×height) pixels, 2 μm per pixel.

### Image analysis of time-lapse development

Embryos were tracked from two synchronised experiments and data pooled for analysis. For Rex1 ESC-injected embryos, one large-sample experiment was analysed. All time-lapse data were analysed manually, using an open-source plug-in for ImageJ (TrackMate). Each time point per embryo was scored for occurrence of death or division. Time and location of these events were recorded throughout. Tracked data was converted from time-steps to minutes; the beginning of each time-lapse movie was offset according to time post-injection, or time after 60 hpc. Hence *t*=0 is defined as 60 hpc. The data from two consecutive experiments performed under identical conditions were pooled.

### Statistical analysis

For each experiment, ESC death and division events were separated across time. The number of viable cells (*V_n_*) was calculated for each embryo at every time point, where a viable cell is defined as one that has not undergone cell death. Hence, at every time point, dead cells were subtracted from the pool of injected cells in each embryo, and newly generated cells were added:
(1)
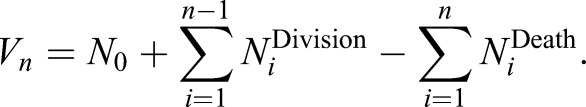
In the case of death of an ESC, the first time point of each experiment was omitted in the cumulative distribution functions (CDFs) as it displayed previous cell death events at unknown time points (thus skewing time distribution inaccurately). The pooled data from the two experiments was separated into five groups based on the number of injected ESCs per embryo (3-7 cells); for Rex1 tracked data, the embryos were placed in one group. For each embryo group, the total numbers of ESC deaths or divisions for each time point were aggregated and normalsed (i.e. an embryo group, containing *n* embryos injected with the same number of ESCs each, contains on average *x*/*n* cell death events in time point *y* per embryo, where *x* is the total number of deaths in *n* embryos at time point *y*). Using a single-embryo profile for the number of accumulated ESC events at each time point, the cumulative distribution function (CDF) was plotted as an average of all embryo groups. To determine whether data points in a set are normally distributed, a one-sample Kolmogorov–Smirnov test was used. When the data points were normally distributed, a two-way *t*-test was used at the 5% significance level. When data points were not normally distributed, a two-way Kolmogorov–Smirnov test was used at the 5% significance level.

### Embryo transfer

Embryos injected with 3-7 tdTomato-H2B ESCs, imaged for 39.5 h were transferred to recipient F1 females rendered pseudopregnant by mating with vasectomised males 2.5 days previously. They were allowed to develop to term and offspring examined for fluorescence.

### Immunohistochemistry

Trypsinised ESCs, collected on glass slides by cytospin, and embryos were prepared for immunohistochemistry as previously described (Kalmar et al., 2009). Primary antibodies were against Nanog (eBiosciences, 14-5761-80), Cdx2 (Cell Signaling Technology, 3977S or Abcam, 157524), Sox17 (R&D, AF1924), Sox2 (BioLegend, 656109 or eBioscience, 14-9811-80), cleaved caspase 3 (Cell Signaling, 9661), Oct4 (Santa Cruz, SC-8628), all used at 1:100 dilution; GFP (Life Technologies, A11122), used at 1:400; c-Myc [Y69] Abcam, ab32072 (lot no. GR184243-1) used at 1:200 dilution according to Sancho et al. (2013). Alexa-Fluor-conjugated secondary antibodies (Molecular Probes) were used at 1:500 dilution. Confocal images were acquired using a Leica TCS SP5 confocal microscope. Images were processed using Leica software, Imaris (Bitplane) and ImageJ (Fiji).

### Quantification of immunofluorescence

For c-Myc experiments, confocal *z*-stacks of images were analysed for intensity of marker expression, either manually (using ImageJ) in 2D for experiments involving small cell numbers (morulae) or semi-automatically in 3D using Volocity (PerkinElmer) software (for experiments requiring high-throughput data analysis). For manual quantification of immunofluorescence, one image plane from each ESC or host nucleus was selected using the ‘Magic Wand’. The option ‘measure’ was selected, which outputs the measurement of the selected area (nuclei) for all channels. For semi-automatic quantification on Volocity, volumes of ESC and host nuclei were identified using the Oct4 channel and appropriate thresholding for partitioning of nuclei. ESCs were separated from host cells by sorting nuclear intensity of the red channel. Volocity measures and outputs all channel intensity for each selected volume. Manual inspection was applied to ensure all parts of nuclei were recorded without overlap between neighbours. Dividing ESCs were excluded from analysis because they displayed no marker expression.
